# Structural polymorphism of the PH domain in TFIIH

**DOI:** 10.1042/BSR20230846

**Published:** 2023-07-13

**Authors:** Masahiko Okuda, Yoshifumi Nishimura

**Affiliations:** 1 Graduate School of Medical Life Science, Yokohama City University, 1-7-29 Suehiro-cho, Tsurumi-ku, Yokohama 230-0045, Japan; 2 Graduate School of Integrated Sciences for Life, Hiroshima University, 1-4-4 Kagamiyama, Higashi-Hiroshima 739-8528, Japan

**Keywords:** budding yeast, general transcription factor, NMR spectroscopy, PH domain, TFIIH

## Abstract

The general transcription factor TFIIH is a multi-subunit complex involved in transcription, DNA repair, and cell cycle in eukaryotes. In the human p62 subunit and the budding yeast *Saccharomyces cerevisiae* Tfb1 subunit of TFIIH, the pleckstrin homology (PH) domain (hPH/scPH) recruits TFIIH to transcription-start and DNA-damage sites by interacting with an acidic intrinsically disordered region in transcription and repair factors. Whereas metazoan PH domains are highly conserved and adopt a similar structure, fungal PH domains are divergent and only the scPH structure is available. Here, we have determined the structure of the PH domain from Tfb1 of fission yeast *Schizosaccharomyces pombe* (spPH) by NMR. spPH holds an architecture, including the core and external backbone structures, that is closer to hPH than to scPH despite having higher amino acid sequence identity to scPH. In addition, the predicted target-binding site of spPH shares more amino acid similarity with scPH, but spPH contains several key residues identified in hPH as required for specific binding. Using chemical shift perturbation, we have identified binding modes of spPH to spTfa1, a homologue of hTFIIEα, and to spRhp41, a homologue of the repair factors hXPC and scRad4. Both spTfa1 and spRhp41 bind to a similar but distinct surface of spPH by modes that differ from those of target proteins binding to hPH and scPH, revealing that the PH domain of TFIIH interacts with its target proteins in a polymorphic manner in Metazoa, and budding and fission yeasts.

## Introduction

The general transcription factor TFIIH functions not only in transcription but also in DNA repair and cell cycle [1–3]. It consists of 10 subunits arranged in two subcomplexes: a Core subcomplex formed by XPB, XPD, p62, p52, p44, p34, and p8; and a CDK-activating kinase (CAK) subcomplex composed of CDK7, Cyclin H, and MAT1 [4,5]. The Core subunits XPB and XPD possess ATP-dependent DNA translocase/helicase activity required for generating either a transcription bubble at transcription start sites or a repair bubble at damaged sites [6,7], allowing TFIIH to scan for a transcription start site (yeast) [8], or perform lesion verification, respectively [9,10]. The CAK subunit CDK7 has kinase activity, which is used for phosphorylating the C-terminal domain of RPB1, the largest subunit of RNA polymerase II, as well as other transcription factors, in order to facilitate the transition from transcription initiation to elongation [11,12]. In nucleotide excision repair (NER), the CAK subcomplex dissociates from the Core subcomplex and is not involved in the repair process [13]; however, it participates in the regulation of cell cycle [14,15].

The N-terminal pleckstrin homology (PH) domain of p62 in the Core subunit is critically involved in recruiting TFIIH to transcription start sites and sites of DNA damage. In recent cryogenic electron microscopy (cryo-EM) structures of the transcription preinitiation alone [16,17] and bound to Mediator [18–20], +1 nucleosome [21,22], and the NER complex [23], the p62 PH domain of TFIIH is invisible. It is also invisible in cryo-EM structures of human TFIIH, indicating that its structure within TFIIH is dynamic [24,25]. Consistent with this, a solution nuclear magnetic resonance (NMR) study of the N-terminal region of p62, comprising the PH domain and the following (BSD1) domain, which is visible in the cryo-EM structure, demonstrated that the high mobility of the interdomain linker is responsible for the dynamic behavior of the PH domain in TFIIH [26].

For this reason, the PH domain, which has a basic surface, is targeted by various transcription and NER factors via an acidic intrinsically disordered region [27–36], referred to as an ‘acidic string’ because it forms an extended string-like conformation when bound to the PH domain. Defects in the interaction between the acidic string and the PH domain lead to a reduction in transcription and NER activities. To date, two NMR structures of the isolated PH domain from human p62 and the budding yeast homologue Tfb1 have been determined [26,37,38]. Similar to the p62 PH domain, the Tfb1 PH domain is targeted by transcription and NER factors [39–46]; however, the target-binding surfaces in each PH domain are compositionally divergent.

In the present study, we have solved the NMR structure of the PH domain derived from fission yeast Tfb1 and have compared it with those of the human and budding yeast PH domains. In addition, we have explored the PH domain-binding site in two Tfb1 target proteins, Tfa1 and Rhp41, and deduced their respective binding surfaces in the fission yeast PH domain by NMR chemical shift perturbation experiments.

## Results

### Design of a construct for the PH domain of fission yeast Tfb1

**Figure 1:**
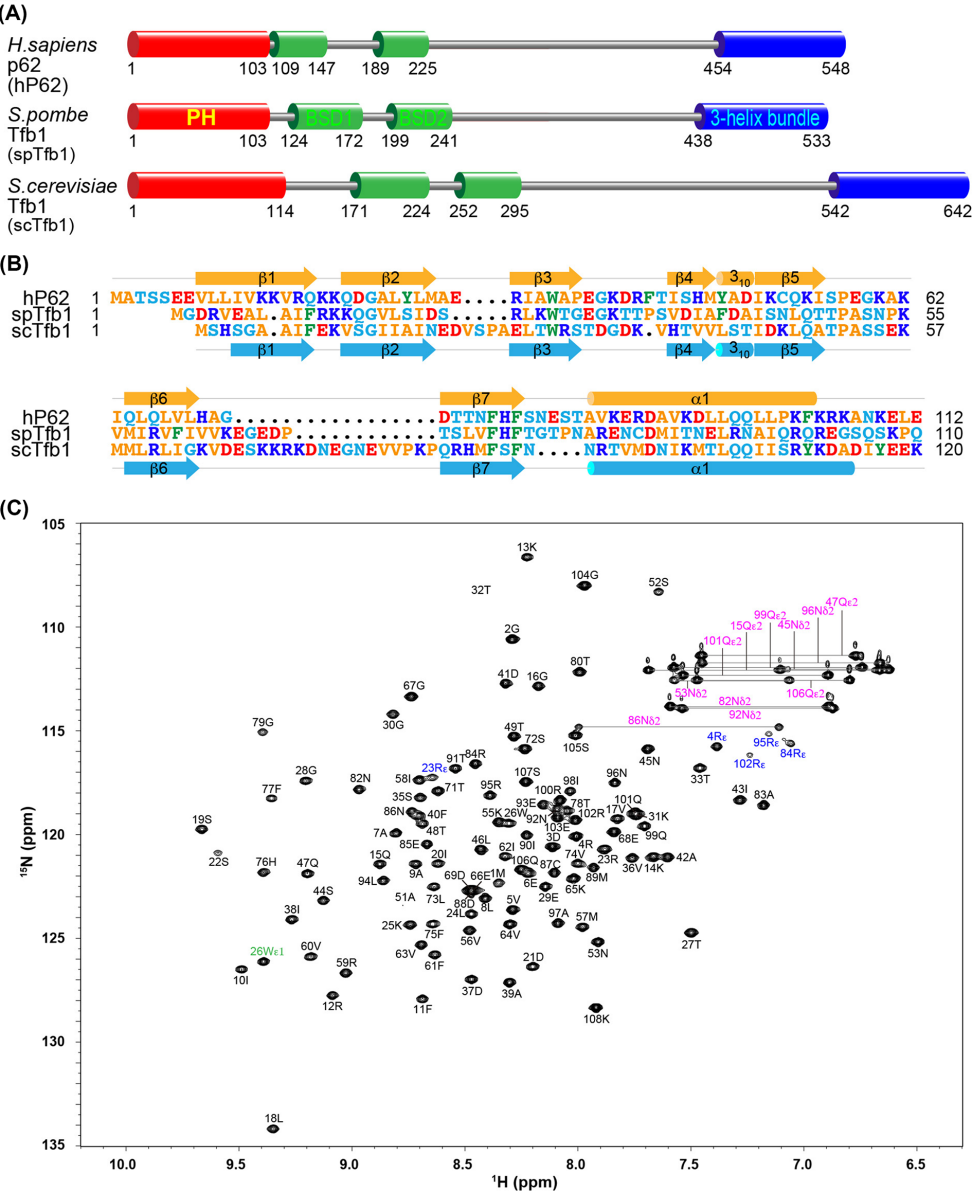
The PH domain of the TFIIH subunit spTfb1 (**A**) Domain organization of the homologous hP62, spTfb1, and scTfb1 subunits. (**B**) Amino acid sequence alignment of the PH domains. Secondary structure elements of the hPH and scPH domains are shown respectively above and below their sequences. Arrow, β-strand; cylinder, helix. (**C**) ^1^H-^15^N-HSQC spectrum of the expressed spPH domain.

While metazoan PH domains are conserved and adopt a similar structure, fungal PH domains are highly divergent in sequence and only the budding yeast structure is available. To gain insight into the functional interactions of the PH domain, we have explored the PH domain from fission yeast Tfb1. Human p62 [UniProt name: TF2H1_HUMAN, General transcription factor IIH subunit 1] (hP62), fission yeast (*Schizosaccharomyces pombe*) Tfb1 [UniProt name: TFB1_SCHPO, General transcription and DNA repair factor IIH subunit tfb1] (spTfb1), and budding yeast (*Saccharomyces cerevisiae*) Tfb1 [UniProt name: TFB1_YEAST, General transcription and DNA repair factor IIH subunit TFB1] (scTfb1) share the same domain organization: an N-terminal PH domain (hereafter designated hPH, spPH, and scPH respectively), two tandem BSD (BTF2-like transcription factors, synapse-associated proteins and DOS2-like proteins) domains, and a C-terminal three-helix bundle, all connected by interdomain linkers (Figure 1A). To design a construct for the spPH domain, we first aligned the N-terminal amino acid sequences of the three proteins (Figure 1B). The spPH domain was predicted to adopt the same secondary structure elements of β-β-β-β-3_10_-β-β-β-α as the hPH and scPH domains. Because the C-terminus of the α1 helix was unclear, we prepared a construct encoding residues 1-108 to allow for the long α1 helix with a small margin. The ^1^H-^15^N-HSQC spectrum of the expressed protein displayed well-dispersed signals, indicating the formation of a properly folded structure (Figure 1C).

### Solution structure of the spPH domain

**Figure 2:**
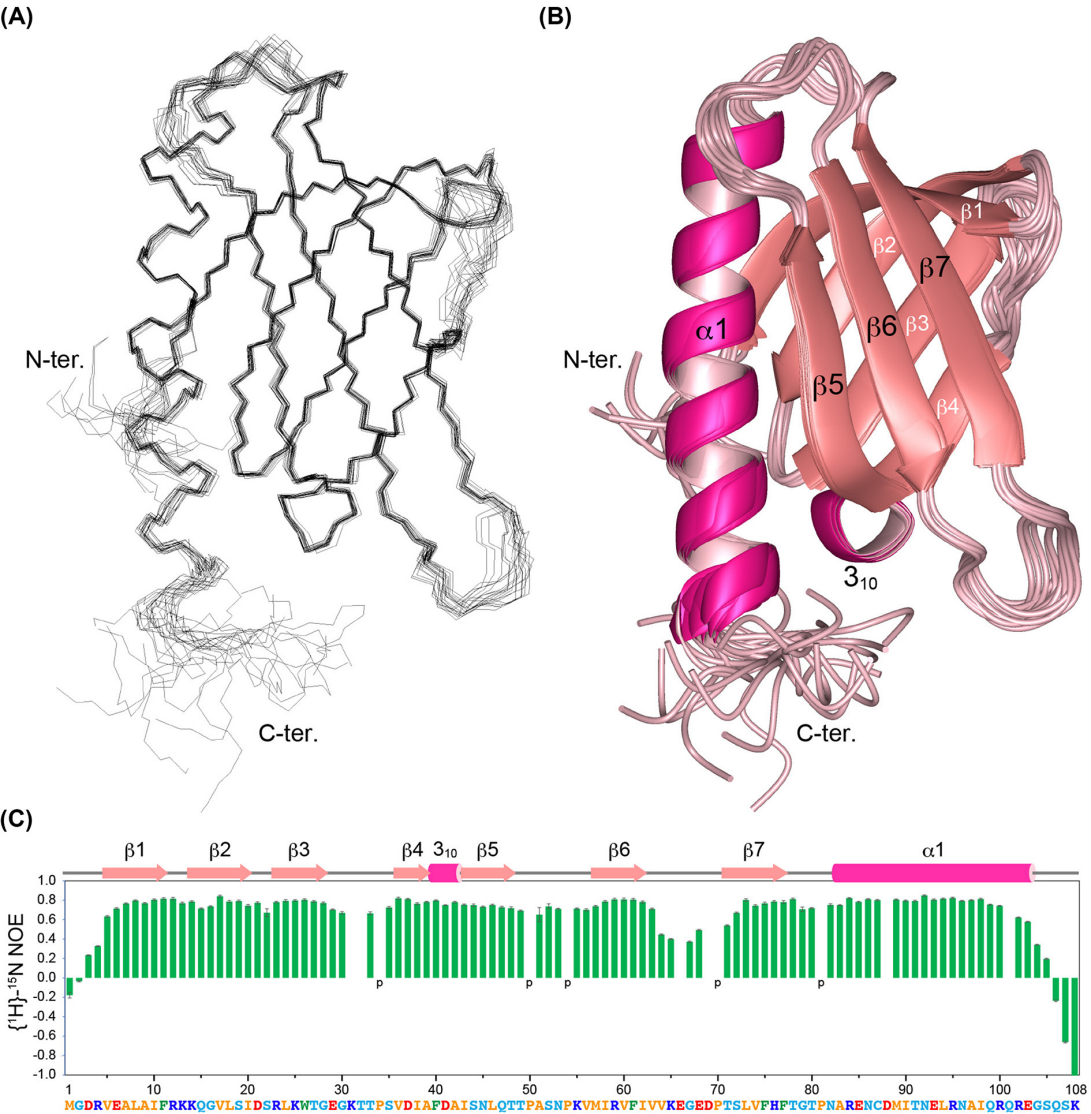
Solution structure of the spPH domain (**A**,**B**) Overlay of the 20 best structures, shown as line (**A**) and ribbon (**B**) diagrams, respectively. (**C**) ^15^N-{^1^H} NOE of the spPH domain. Proline residues are indicated by ‘p’. For some residues, no NOE value was determined due to an overlapping or unreliable weak amide peak.

We explored the structural features of the spPH domain by determining its solution structure by NMR (Table 1 and Figure 2A,B). Two antiparallel β-sheets, consisting of strands β1–β4 and β5–β7, form a β-barrel-like structure, in which the discontinuity between strands β4 and β5 is compensated by a one-turn 3_10_-helix and the open space between strands β1 and β5 is packed with a six-turned α1 helix, making a compact globular structure (Figure 2B). A heteronuclear ^15^N-{^1^H} NOE experiment showed that three small regions—the N-terminal four residues, residues 64–68 between strands β6 and β7, and the C-terminal five residues—displayed high mobility with ^15^N-{^1^H} NOE values lower than 0.5, indicating a relatively rigid structure (Figure 2C). The secondary structure elements were ultimately defined as follows: β1, residues 5–11; β2, 14–20; β3, 23–28; β4, 36–39; 3_10_-helix, 40–42; β5, 43–48; β6, 57–62; β7, 71–77; and α1 helix, 83–103 (Supplementary Figure S1A). There was a small but significant difference in backbone and heavy atoms between the NMR structure and the structure predicted by AlphaFold [47] (Supplementary Figure S2).

**Table 1:** Statistics for the 20 best structures of TFIIH spPH domain

Experimental restraints	
Total NOE	2627
Intraresidue	313
Sequential (i-j = 1)	685
Medium-range (1<i-j<5)	404
Intramolecular long-range (i-j≥5)	1225
Hydrogen bond	42x2
Number of dihedral restraints	
φ	75
ψ	76
χ1	58
χ2	6
Statistics for structure calculations	
R.m.s. deviations from experimental restraints^*^	
Distance (Å)	0.037 ± 0.001
Dihedral (°)	0.435 ± 0.041
R.m.s. deviations from idealized covalent geometry	
Bonds (Å)	0.00569 ± 0.00016
Angles (°)	0.732 ± 0.018
Improper (°)	0.764 ± 0.019
Coordinate precision	
Average pairwise r.m.s. deviation from the mean structure	
Backbone atoms (Å)	0.56 ± 0.11^†^
Heavy atoms (Å)	1.28 ± 0.18^†^
Ramachandran plot statistics	
Residues in most favored regions (%)	82.2^‡^
Residues in additional allowed regions (%)	15.4^‡^
Residues in generously allowed regions (%)	1.5^‡^
Residues in disallowed regions (%)	1.0^‡^

* None of the structures exhibited distance violations > 0.5 Å, dihedral angle violations > 5°.

† The value was calculated over residues 4–102.

‡The value was calculated over residues 1-108.

### Comparison of backbone structure between the spPH domain and the hPH and scPH domains

**Figure 3:**
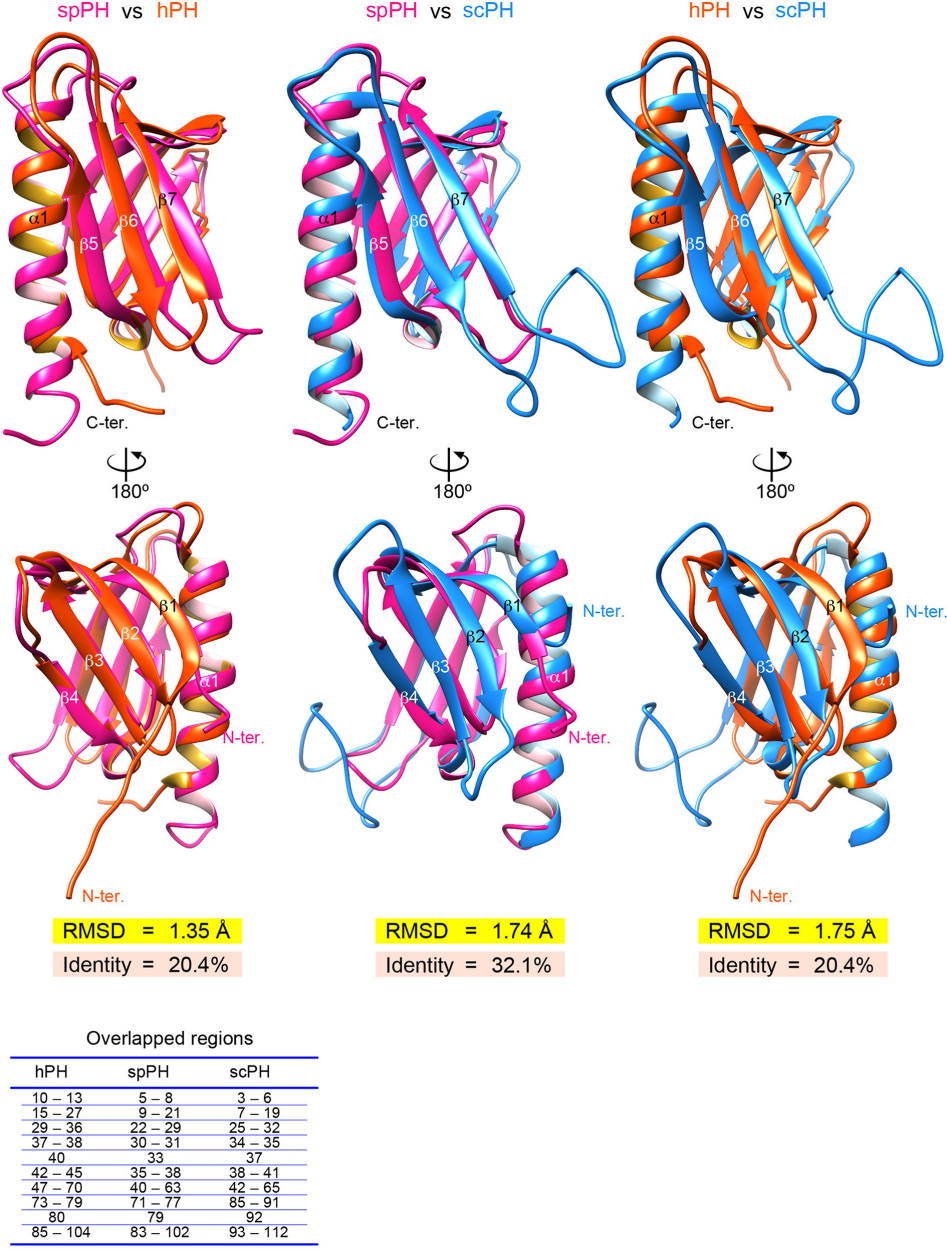
Comparison of the backbone structures of the hPH, spPH, and scPH domains hPH domain is colored orange-red, spPH domain deep pink, and scPH domain blue. Pairwise RMSD and amino acid sequence identity are indicated. The overlapped regions are listed below.

Next, we compared the structure of the spPH domain with those of the hPH and scPH domains (Supplementary Figure S1B). To assess similarity between the backbone structures, pairwise root-mean-square deviations (RMSDs) of the backbone atoms (N, Cα, C, and O) in the superimposed core regions (84 residues in total) were calculated (Figure 3). The RMSDs for pairwise comparisons of the spPH and hPH, spPH and scPH, and hPH and scPH domains were 1.35 Å, 1.74 Å, and 1.75 Å, respectively, while the respective amino acid sequence identities were 20.4%, 32.1%, and 20.4%. We identified 33 residues critical for formation of the hydrophobic core of the spPH domain (Supplementary Figure S3). In the hydrophobic region (57 residues in total), the pairwise backbone RMSDs of the spPH and hPH, spPH and scPH, and hPH and scPH domains were 1.05 Å, 1.38 Å, and 1.36 Å, and the amino acid sequence identities were 17.5%, 31.6%, and 21.1%, respectively. Thus, with respect to the backbone structure of the overlapped core regions of the PH domain, fission yeast is more similar to human than to budding yeast, in contrast with amino acid sequence identity.

Although the three PH domains adopt a similar core structure, beyond the core they have different tail and turn structures (Figure 3 and Supplementary Figure S1). Strands β2 and β3 are connected by two residues in spPH and hPH, but by six residues in scPH. Strands β6 and β7 are connected by eight residues in spPH, which is longer than in hPH (three residues), and shorter than in scPH (20 residues). Strand β7 and the α1 helix are linked by five residues in spPH and hPH, but by only one residue in scPH. In summary, the regions outside the core in the fission yeast PH domain are structurally intermediate between those of the human and budding yeast PH domains, and more similar to the human PH domain than to the budding yeast PH domain.

### Predicted target-binding surface of the spPH domain

**Figure 4:**
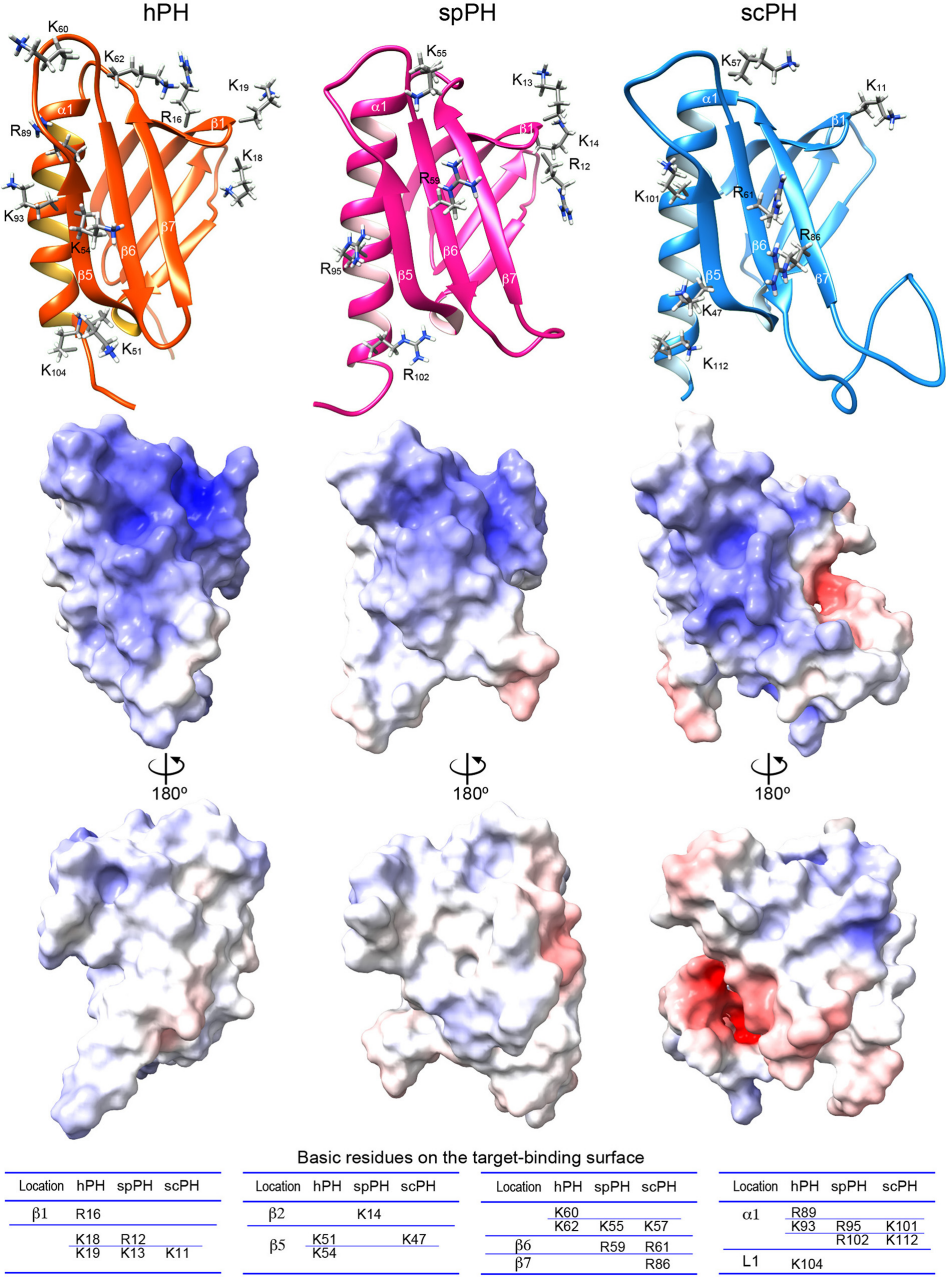
Comparison of the electrostatic potential surfaces of the hPH, spPH, and scPH domains Upper panel, backbone structure with side-chains of basic amino acids involved in target binding. Middle and lower panels, electrostatic potential surface. Positive potential is shown in blue, and negative potential in red. Residues that form the basic surface are listed below.

The available structures of complexes of the hPH and scPH domains with target proteins have identified common modes of recognition [28–30,32–36,39–46]. In brief, all target proteins bind to the PH domain through a highly acidic intrinsically disordered region that interacts electrostatically with the widely distributed basic residues and inserts residues into two pockets in the PH domain. The spPH domain adopts a basic surface in a similar position to the hPH and scPH domains (Figure 4, middle panel); however; the distribution of positive potential is distinct, owing to the different positioning of several basic residues. On strand β5, for example, there are no basic residues in spPH, two (Lys51 and Lys54) in hPH, and one (Lys47) in scPH; on strand β6, there is one basic residue (Arg59) in spPH, none in hPH, and one (Arg61) in scPH; and on strand β7, there are no basic residues in spPH and hPH, and one (Arg86) in scPH.

**Figure 5:**
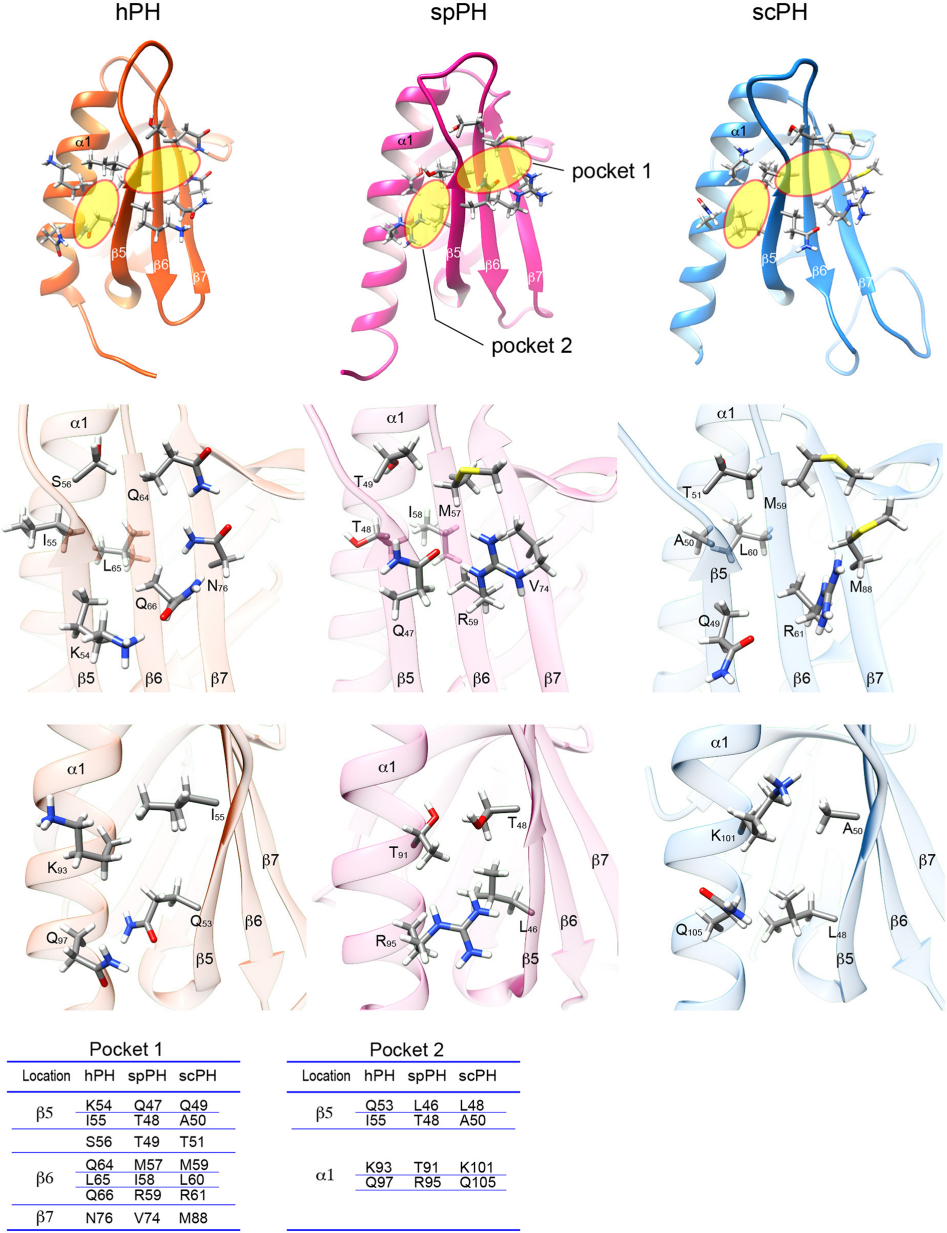
Comparison of the target-binding pockets of the hPH, spPH, and scPH domains Upper panel, target-binding pockets 1 and 2 (yellow circles). Middle panel, close up of pocket 1. Lower panel, close up of pocket 2. Residues that form the respective pockets are listed below.

The two target-binding pockets in the hPH and scPH domains are designated ‘pocket 1’ and ‘pocket 2’. The corresponding pockets are also seen in the spPH domain (Figure 5). Pocket 1 is formed by Gln47, Thr48, and Thr49 on strand β5; Met57, Ile58, and Arg59 on strand β6 strand; and Val74 on strand β7 (Figure 5, middle panel). Pocket 2 is formed by Leu46 and Thr48 on strand β5, and Thr91 and Arg95 on the α1 helix (Figure 5, lower panel). A comparison of these pockets with those in other PH domains shows that a variety of amino acids contribute to pocket formation.

### Binding activity of the spPH domain

**Figure 6:**
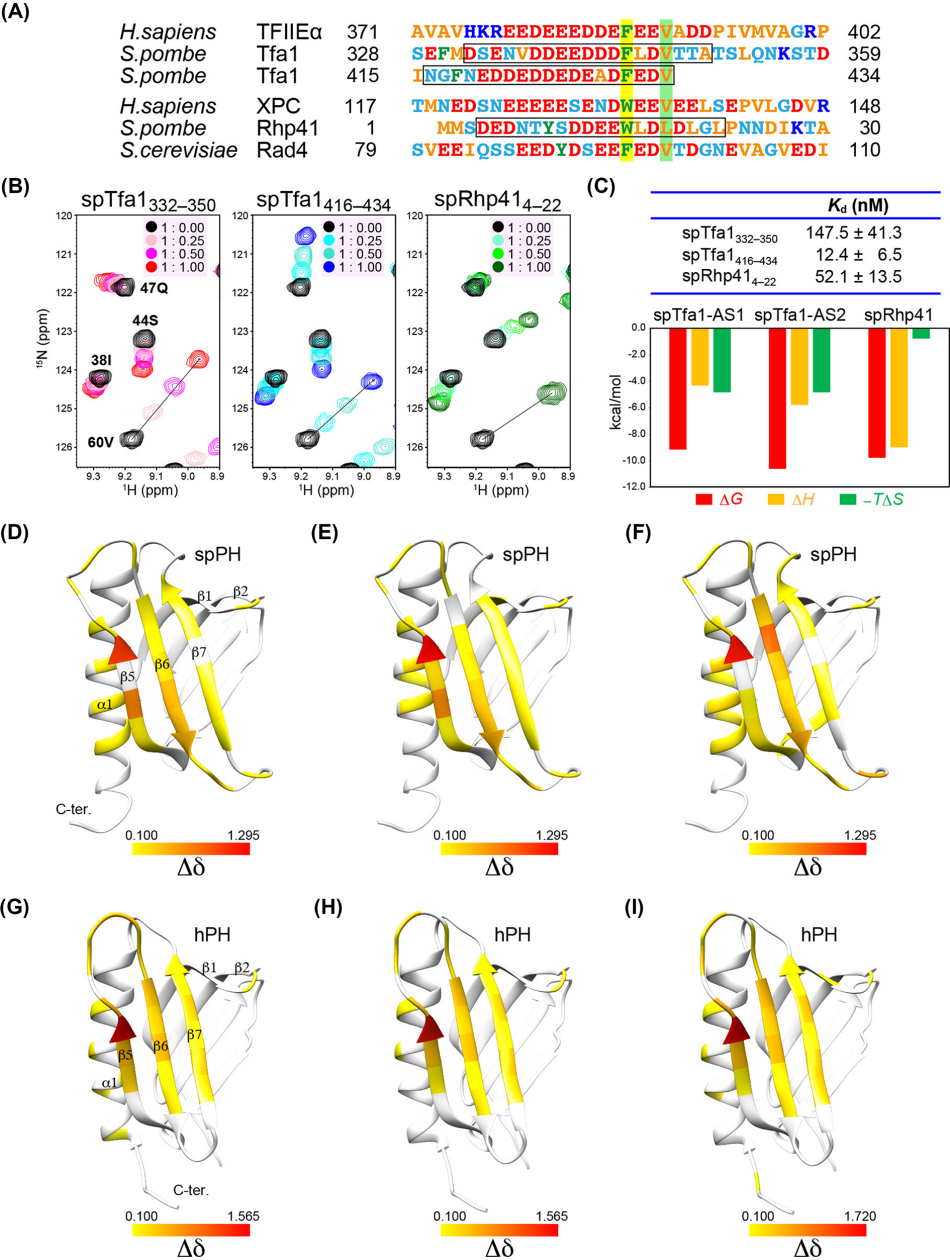
Target-binding activity and the deduced binding surface of the spPH domain (**A**) Amino acid sequence alignment of p62/Tfb1 target proteins. Pocket 1- and 2-inserted residues of hTFIIEα, hXPC, and scRad4, and the corresponding residues in other target proteins, are highlighted in yellow and light green, respectively. The sequences of peptides used in chemical shift perturbation (CSP) are indicated by boxes. (**B**) NMR CSP experiment. Overlay of four ^1^H-^15^N-HSQC spectra of the spPH domain alone (black) and titrated with target peptide at molar ratios of 1:0.25, 1:0.50, and 1:1.00. Selected regions are shown here (see Supplementary Figure S4 for the full regions). (**C**) Estimated *K*_d_ values and thermodynamic parameters. (**D**-**I**) CSP mapping. Residues showing a chemical shift change (Δδ) greater than 0.100 upon addition of the target peptide are mapped on the structure of the PH domain: spTfa1_332-350_ (**D**); spTfa1_416-434_ (**E**); spRhp41_4-22_ (**F**); hTFIIEα_378-439_ (acidic domain) (**G**); hTFIIEα_378-396_ (**H**); and hXPC_109-156_ (**I**). Residues are colored according to the magnitude of Δδ.

Next, we examined the binding activity of the spPH domain for two target proteins: a general transcription factor and an NER factor. The human general transcription factor TFIIEα binds to the hPH domain by mainly two regions of the acidic domain: a 16-residue N-terminal tail and a five-residue C-terminal tail [27,28]. Amino acid sequence alignment suggested that the fission and budding yeast Tfa1 homologues have no structure corresponding to the core of the TFIIEα acidic domain; interestingly, however, fission yeast Tfa1 has two acidic segments that are similar to the N-terminal tail (acidic string) of the TFIIEα acidic domain (Figure 6A) [28], but how these regions interact with the PH domain remains unclear. We therefore investigated the binding ability of the two acidic segments, residues 332-350 (spTfa1_332-350_) and 416-434 (spTfa1_416-434_) of fission yeast Tfa1 (spTfa1), by an NMR chemical shift perturbation (CSP) experiment. Addition of the spTfa1_332-350_ peptide and the spTfa1_416-434_ peptide markedly changed a subset of signals of the spPH domain (Figure 6B and Supplementary Figure S4). In addition, ITC experiments demonstrated that the binding affinity of the spTfa1_416-434_ peptide was stronger than that of the spTfa1_332-350_ peptide: dissociation constant (*K*_d_) values for the interaction between the spPH domain and the spTfa1_332-350_ and the spTfa1_416-434_ were 147.5 ± 41.3 and 12.4 ± 6.5 nM, respectively (Figure 6C). Thus, the main binding site of spTfa1 was found to be in the C-terminal acidic segment.

In global genome NER, the human DNA lesion sensor protein XPC (hXPC) recruits TFIIH to lesions through its interaction with the p62 and XPB subunits [48]. Importantly, the interaction between the acidic string of hXPC and the hPH domain of p62 plays a predominant role in the recruitment of TFIIH to sites of damage [30]. The importance of this interaction between the budding yeast counterparts—namely, the acidic string of Rad4 (scRad4) and the scPH domain in NER—has been verified [43]. Here, we identified a sequence in the fission yeast homologue Rhp41 that may fulfil the common rules for recognition of the PH domain (residues 4-22, spRhp41_4-22_) (Figure 6A). Unlike hXPC and scRad4, the binding site is located at the N-terminus of Rhp41. It contains a tryptophan (Trp15) in the acidic sequence, corresponding to Trp133 of hXPC and Phe95 of scRad4, each of which occupies pocket 1 in their respective PH domain; however, it does not contain a valine equivalent to Val136 of hXPC or Val98 of scRad4, which occupies pocket 2 in their respective PH domain. Instead, the binding site of Rhp41 has a similar hydrophobic residue, leucine (Leu18), and uniquely, there are four leucine residues at regular intervals in this region (Leu16, Leu18, Leu20, and Leu22). In the NMR CSP experiment, some specific signals of the spPH domain were markedly altered on the addition of spRhp41_4-22_ peptide corresponding to this region (Figure 6B and Supplementary Figure S4) and *K*_d_ for the interaction between the spPH domain and the spRhp41_4-22_ peptide was found to be 52.1 ± 13.5 nM by ITC (Figure 6C).

### Target-binding surfaces of the spPH domain

To deduce the spTfa1- and spRhp41-binding surfaces, we mapped residues showing large chemical shift changes on the structure of the spPH domain (Figure 6D–F). In common to both the spTfa1 and spRhp41 titrations, these residues were mapped on the turn between strands β1 and β2, the second antiparallel β-sheets (strands β5-β7), the loops between strands β5 and β6 and strands β6 and β7, and the middle part of the α1 helix. The deduced surface for binding spTfa1_416-434_ in the spPH domain (Figure 6E) is similar to that estimated for binding the hTFIIEα acidic domain in the hPH domain for both the whole acidic domain (Figure 6G) and the N-terminal acidic string peptide (Figure 6H) [28,33]. Given that the spTfa1 peptide binds to the spPH domain in the same direction as the N-terminal acidic string of the hTFIIEα acidic domain, no or only a small CSP on the C-terminal part of the α1 helix on addition of spTfa1_416-434_ peptide would result from its shortness at the C-terminus. The deduced spRhp41_4-22_-binding surface (Figure 6F) is similar to the spTfa1-binding surface (Figure 6D,E). It also resembles the hXPC-binding surface in the hPH domain (Figure 6I) [30].

## Discussion

### Polymorphism of the PH domain in TFIIH

**Figure 7:**
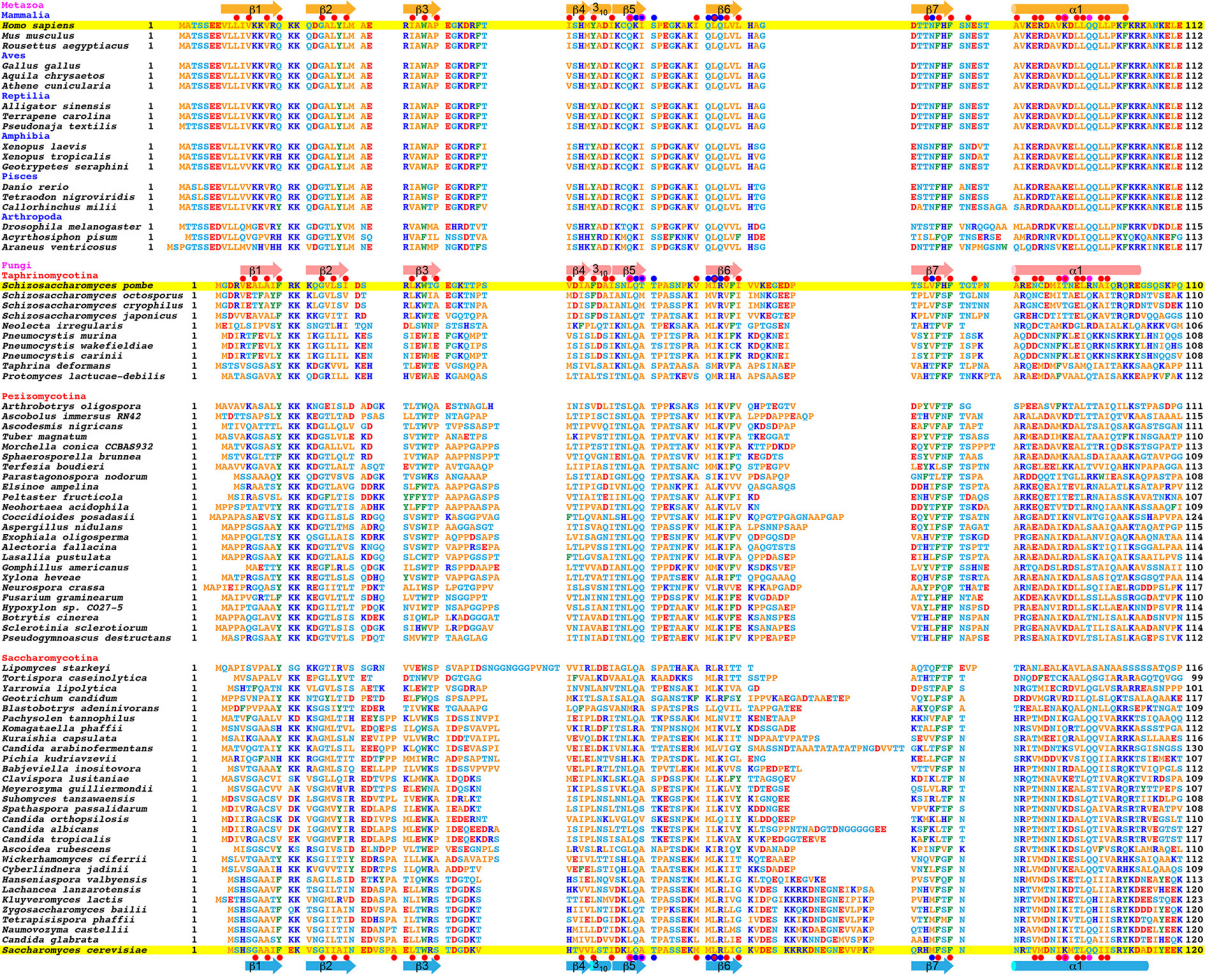
Amino acid sequence alignment of metazoan p62 and fungal Tfb1 PH domains Secondary structure elements of the hPH, spPH, and scPH domains are shown above and below their sequences, which are highlighted in yellow. Arrow, β-strand; cylinder, helix. Red dots, residues that form a hydrophobic core; blue dots, residues that form pocket 1; magenta dots, residues that form pocket 2.

In human and budding yeast, the PH domain of the p62/Tfb1 subunit dynamically guides TFIIH to transcription-start sites and DNA-damage sites through its interactions with transcription and NER factors containing a specific acidic string. The hPH domain shares a marked degree of amino acid sequence similarity with other metazoan PH domains (Figure 7); thus, the structures of metazoan PH domains will be essentially identical to or may be precisely predicted from the available structure of hPH. By contrast, this striking sequence similarity is not seen among fungi, although the structure of the PH domain from budding yeast is available. Fission yeast is widely used as a model for biological processes in human cells. It belongs to a different subphylum (Taphrinomycotina) from budding yeast (Saccharomycotina) (Figure 7), and its PH domain has a few differences in amino acid sequence relative to budding yeast. The present study has revealed that the spPH domain is more similar to the hPH domain than to the scPH domain with respect to the backbone structure of the superimposed core region including the hydrophobic core, regardless of its higher sequence similarity to the scPH domain. The regions outside the core also show closer resemblance to the hPH domain than to the scPH domain. Although fungal PH domains are apparently divergent, many residues that form the hydrophobic core are generally conserved in each subphylum (Figure 7). In addition, regions connecting the secondary structure elements are similar in length in each subphylum. Thus, the structural similarities and differences present among the hPH, spPH, and scPH domains are likely to be generally applicable to a wide range of species.

**Figure 8:**
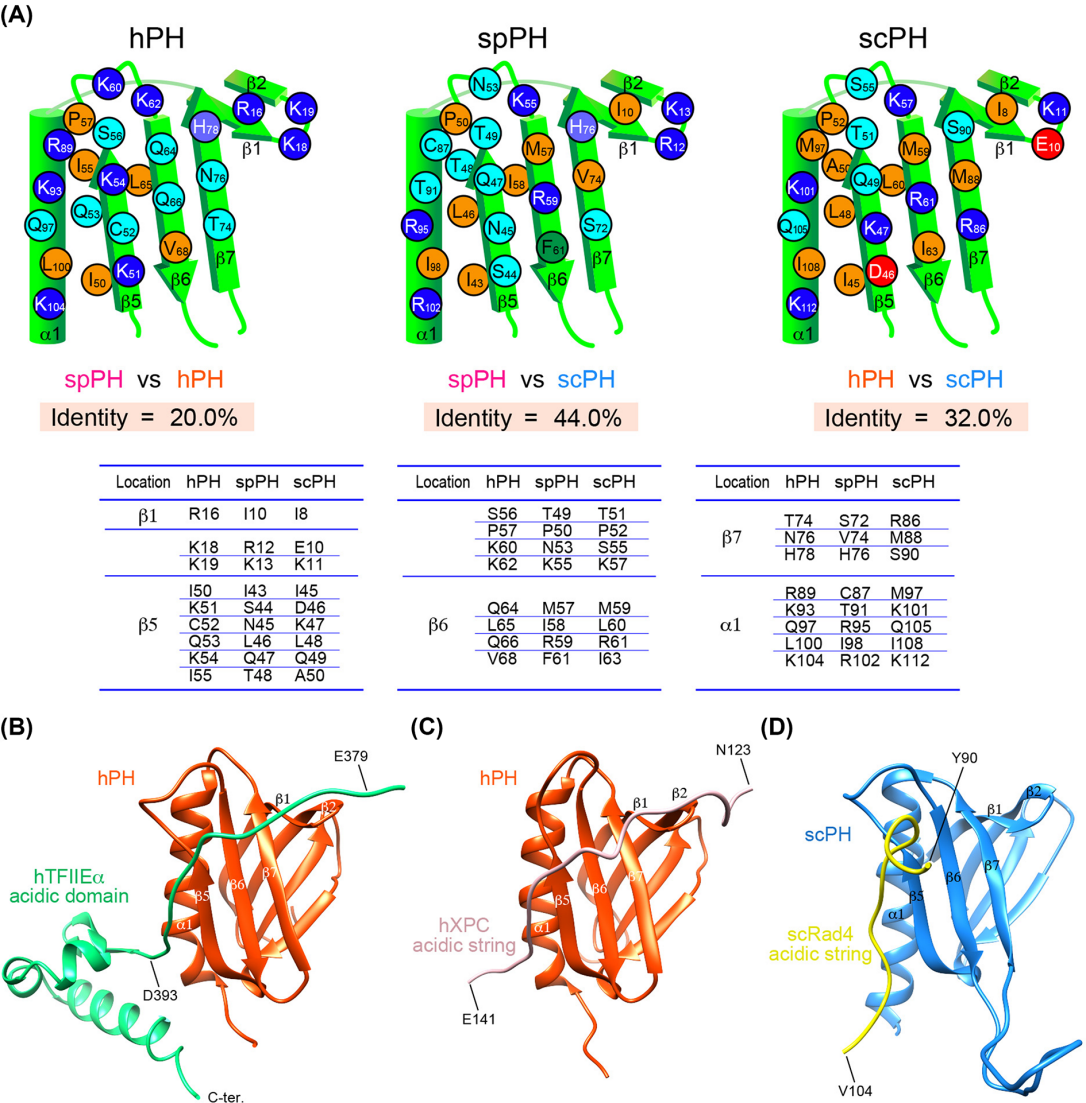
Comparison of the target-binding surfaces of the hPH, spPH, and scPH domains (**A**) Schematic target-binding surfaces of the PH domains. Pairwise amino acid identity is indicated. Residues that form the target binding surface and the corresponding residues are listed below. (**B**-**D**) Structures of the complex of the PH domain and its target. (**B**) Complex of the hPH domain (orange-red) with the hTFIIEα acidic domain (green) [PDB ID 2RNR]. (**C**) Complex of the hPH domain (orange-red) with the hXPC acidic string (pale pink) [PDB ID 2RVB]. (**D**) Complex of the scPH domain (blue) with the scRad4 acidic string (yellow) [PDB ID 2M14].

Regarding the target-binding surface of the PH domain, the amino acid identity between the spPH and hPH, spPH and scPH, and hPH and scPH domains is 20.0%, 44.0%, and 32.0%, respectively, indicating that the composition of the target-binding surface of spPH more closely resembles that of the scPH domain than that of the hPH domain (Figure 8A). The spPH domain contains the same pocket-1-forming residues (Gln47, Thr49, Met57, and Arg59) as the scPH domain (Gln49, Thr51, Met59, and Arg61); these residues closely interact with a key aromatic residue in their target proteins and are highly conserved among yeasts, suggesting a similar mode of binding. However, the spPH domain lacks a residue identified as critical for binding in the scPH domain. Namely, Lys47 on strand β5 is predicted to form a potential salt bridge with an acidic residue located at the second position after the pocket-1-inserted aromatic amino acid of the acidic string of target proteins such as the NER factors Rad2 and Rad4 [42,43]. The corresponding residue in the spPH domain is Asn45. The lysine residue in the scPH domain is not widely conserved in yeasts; therefore, it seems to be uniquely important to a subset of yeasts in the Saccharomycotina subphylum (Figure 7).

Notably, the spPH domain contains residues identified as necessary for binding by the hPH domain. For example, Lys18 in the turn between strands β1 and β2 of the hPH domain consistently makes electrostatic interactions with acidic amino acids located in the N-terminal region of the acidic string of target proteins [27–36]. The corresponding residue is the same basic residue (Arg12) in the spPH domain but is an acidic residue (Glu10) in the scPH domain (Figure 8A). As in the case of Lys47, the acidic residue at this position is limited to a subset of yeasts in the Saccharomycotina subphylum (Figure 7), while the basic residue at this position is seen in a wide range of species. Consistent with this, complexity and diversity are seen between the acidic strings of the homologous target proteins—namely, hTFIIEα and spTfa1, and hXPC, spRhp41, and scRad4—as discussed below.

### Complexity and diversity of the TFIIH PH domain’s target proteins

By examining the binding activity of the spPH domain, we found that the interactions of the hPH domain with both hTFIIEα and hXPC are conserved in fission yeast. In terms of the PH domain-binding site, however, hTFIIEα has an acidic domain containing the acidic string, and this acidic domain binds to the hPH domain with a *K*_d_ value of 95 nM (Figure 8B) [28,30]. In contrast, spTfa1 seems to have two binding strings: namely, a lower affinity (*K*_d_ = 148 nM) segment and a higher affinity (*K*_d_ = 12 nM) segment with no structured domain between them (Figure 6A). The main binding segment, spTfa1_416-434_, contains a strong acidic string comprising _420_EDDEDDEDEADFEDV_434_, while the corresponding sequence of spTfa1_332-350_ is _333_SENVDDEEDDDFLDV_347_. The different acidity between two strings may contribute the variation in binding ability; however, the molecular mechanisms and reasons for the different binding affinities of two spTfa1 acidic strings should be investigated in future studies.

Unlike hTFIIEα, hXPC and scRad4 bind to the PH domain by their continuous acidic strings; however, the hXPC-binding surface in the hPH domain is wider than the scRad4-binding surface in the scPH domain (Figure 8C,D). In this regard, the deduced spRhp41-binding surface is more similar to the hXPC-binding surface, because Arg12 and Lys13 on the turn between strands β1 and β2 in the spPH domain displayed substantial chemical shift changes in the NMR CSP experiment (Figure 6F). The corresponding Lys18 and Lys19 residues of the hPH domain electrostatically interact with multiple acidic residues in the N-terminal region of the acidic string of hXPC (Figure 8C) [30]. In contrast, the corresponding Glu10 and Lys11 residues of the scPH domain are positioned far from the acidic string of scRad4 in the complex structure (Figure 8D) [43]. Interestingly, however, the acidic strings of these different proteins bind to their PH domains with a similar affinity: spRhp41, *K*_d_ = 52 nM; hXPC, *K*_d_ = 58 nM [30]; and scRad4, *K*_d_ = 50 nM [43].

The detailed recognition mechanisms underlying the interaction between the spPH domain and the acidic strings of spTfa1, spRhp41, and other target proteins will be elucidated from structures of these complexes in future studies. Furthermore, a structural comparison of fission yeast complexes with human and budding yeast ones will rationalize the complexity and diversity observed in both the target-binding surface of the PH domains and the acidic strings of target proteins, and will uncover structural principles of PH domain recognition that are commonly present throughout all species, as well as features that have become specialized in respective organisms.

## Materials and methods

### Preparation of the spPH domain

The ^15^N- or ^13^C/^15^N-labeled spPH domain (residues 1-108) was prepared by a previously described method [28]. In brief, the spPH domain was expressed as a hexa-histidine-tagged product in a pET15b vector (Merck Millipore) in *Escherichia coli* BL21-Gold (DE3) (Agilent Technologies). The lysed supernatant was loaded on to a cOmplete His-Tag purification resin column (Roche), and the eluate was digested with thrombin (Cytiva) to remove the histidine tag. After concentration with an Amicon Ultra device (Merck Millipore), the sample was purified on a Superdex75 column (GE Healthcare).

### NMR structure determination

For structure determination, we used 0.9–1.0 mM ^15^N- and ^13^C/^15^N-labeled spPH domain in 20 mM potassium phosphate (pH 6.8) and 5 mM deuterated DTT, prepared in either 90% H_2_O/10% D_2_O or 99.9% D_2_O. NMR experiments were performed at 25°C on AVANCE III HD 600 MHz and 950 MHz spectrometers (Bruker), each equipped with a Cryo-TCI probe. Backbone and side-chain resonances were assigned by using standard triple-resonance NMR experiments [49]. Stereospecific assignments were obtained from a combination of HNHB, HN(CO)HB, HNCG, HN(CO)CG, and ^13^C-edited and ^15^N-edited NOESY-HSQC spectra. Distance restraints were obtained from ^15^N-edited NOESY-HSQC (τ_m_ = 50, 150 ms) and ^13^C-edited NOESY-HSQC (τ_m_ = 50, 100 ms) spectra. Side-chain torsion angles, χ1 and χ2, were obtained from a combination of HNHB, HN(CO)HB, HNCG, HN(CO)CG, and ^13^C-edited and ^15^N-edited NOESY-HSQC spectra. Hydrogen bond donors were estimated by backbone amide H/D-exchange experiments, and their acceptors were ultimately determined based on the final structure. Hydrogen bond restraints were used during the refinement stage of calculation. Spectra were processed by using NMRPipe [50], and analyzed by using NMRView [51] and Magro [52].

### Structure calculation

Interproton distance restraints derived from NOE intensities were grouped into four distance ranges corresponding to strong, medium, weak, and very weak NOEs: 1.8–2.7 Å (1.8–2.9 Å for NOEs involving HN protons), 1.8–3.3 Å (1.8–3.5 Å for NOEs involving HN protons), 1.8–5.0, and 1.8–6.0 Å, respectively. The upper limit was corrected for constraints involving methyl groups, aromatic ring protons, and non-stereo-specifically assigned methylene protons. Dihedral angle restraints for φ and ψ were obtained from analysis of the backbone chemical shifts with TALOS+ [53]. χ1 and χ2 angles were restrained ± 30° for three side-chain rotamers. Structure calculations were performed by distance geometry and simulated annealing using the program Xplor-NIH [54,55]. In total, we calculated 100 structures, which were each subjected to water refinement [56]. Statistics for the 20 best structures are summarized in Table 1. Structures were also calculated without using hydrogen bond restraints (Supplementary Figure S5). Structures were analyzed and displayed by using PROCHECK-NMR [57], MOLMOL [58], CHIMERA [59], and PyMol (http://www.pymol.org).

### Heteronuclear NOE experiment

To determine steady-state ^15^N-{^1^H} NOE values, the ^15^N-labeled spPH domain was probed at 25°C on an AVANCE III HD a 600-MHz spectrometer (Bruker) equipped with a Cryo-TCI probe. NOE values were determined from peak intensity ratios obtained from spectra acquired with and without proton saturation. Uncertainties were determined from the standard deviation in background noise levels calculated by using NMRView [51].

### NMR chemical shift perturbation

Each spTfa1_332-350_, spTfa1_416-434_, or spRhp41_4-22_ peptide (GenScript) was added to 0.1 mM ^15^N-labeled spPH domain at molar ratios of 1:0.00, 1:0.25, 1:0.50, and 1:1.00 in 20 mM potassium phosphate (pH 6.8), 100 mM NaCl, 5 mM deuterated DTT, and 10% D_2_O. ^1^H-^15^N-HSQC spectra were acquired before and after the addition of peptide samples at 25°C on an AVANCE III HD 600 MHz spectrometer (Bruker) equipped with a Cryo-TCI probe. Spectra were processed by NMRPipe [50], and analyzed by NMRView [51]. The chemical shift change (Δδ) was calculated as Δδ = {(Δδ^1^H)^2^ + (Δδ^15^N/5)^2^}^1/2^. Residues showing chemical shift changes were mapped on the protein surface by CHIMERA [59].

### ITC

The *K*_d_ values were measured by ITC using a VP-ITC calorimeter (Micro-Cal). The syringe contained 300 μM spTfa1 or spRhp41 peptide; the cell contained 2 ml of 30 μM spPH domain. Titrations (25 × 20 μl injections) were carried out in 20 mM potassium phosphate (pH 6.8) at 20°C. Each injection took 4 s, with a pre-injection delay of 210 s and a syringe stirring speed of 307 rpm. Data were analyzed by using the Origin software package (MicroCal).

## Supplementary Material

Supplementary Figures S1-S5

## Data Availability

The coordinate of the spPH domain has been deposited in the RCSB PDB with code 8I53. NMR resonance assignments and steady-state ^15^N-{^1^H} NOE values of the spPH domain have been deposited in the Biological Magnetic Resonance Bank with code 36546.

## Abbreviations

BSD: BTF2-like transcription factors, synapse-associated proteins and DOS2-like proteins
CAK: CDK-activating kinase
cryo-EM: cryogenic electron microscopy
CSP: chemical shift perturbation
NER: nucleotide excision repair
NMR: nuclear magnetic resonance
PH: pleckstrin homology
RMSD: root-mean-square deviation
sc: *Saccharomyces cerevisiae*
sp: *Schizosaccharomyces pombe*
