# Supplementary material for: Structural polymorphism of the PH domain in TFIIH
Source: Biosci Rep. 2023 Jul 13;43(7):BSR20230846. doi: 10.1042/BSR20230846 (PMC10345426; doi:10.1042/BSR20230846)
Supplement: Supplementary Figures S1-S5 [file BSR-2023-0846_supp.pdf]

Supplementary Figures

Structural polymorphism of the PH domain in TFIIH

Masahiko Okuda and Yoshifumi Nishimura

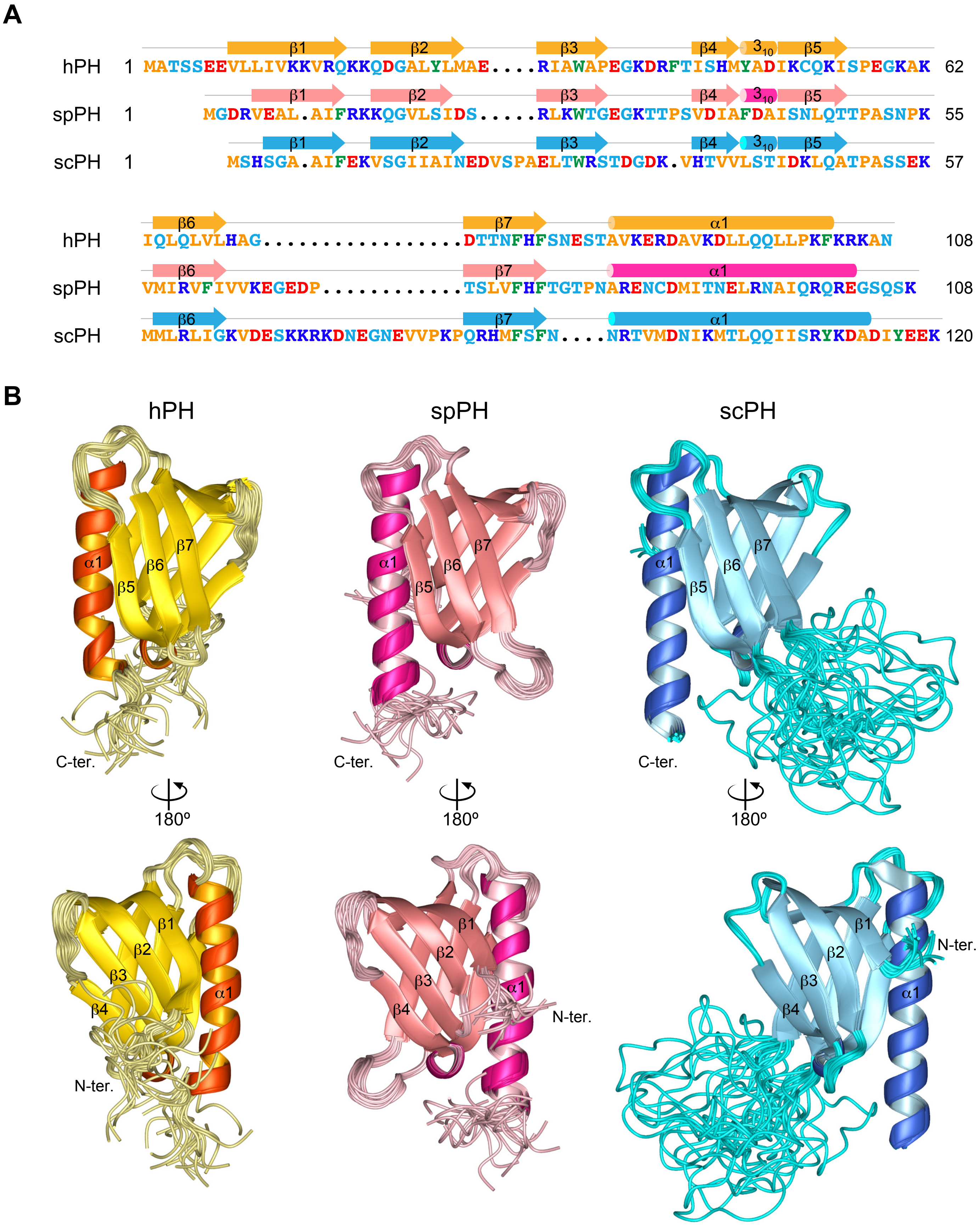

Supplementary Figure 1. Structural comparison of the hPH, spPH, and scPH domains.

(**A**) Amino acid sequence alignment of the three PH domains. Secondary structure elements are shown above each sequence. Arrow, β-strand; cylinder, helix.

(**B**) Twenty best NMR structure ensembles of the PH domains.
hPH domain is colored orange-red [PDB ID 7BUL], spPH domain deep pink [PDB ID 8I53], and scPH domain blue [PDB ID 1Y5O].

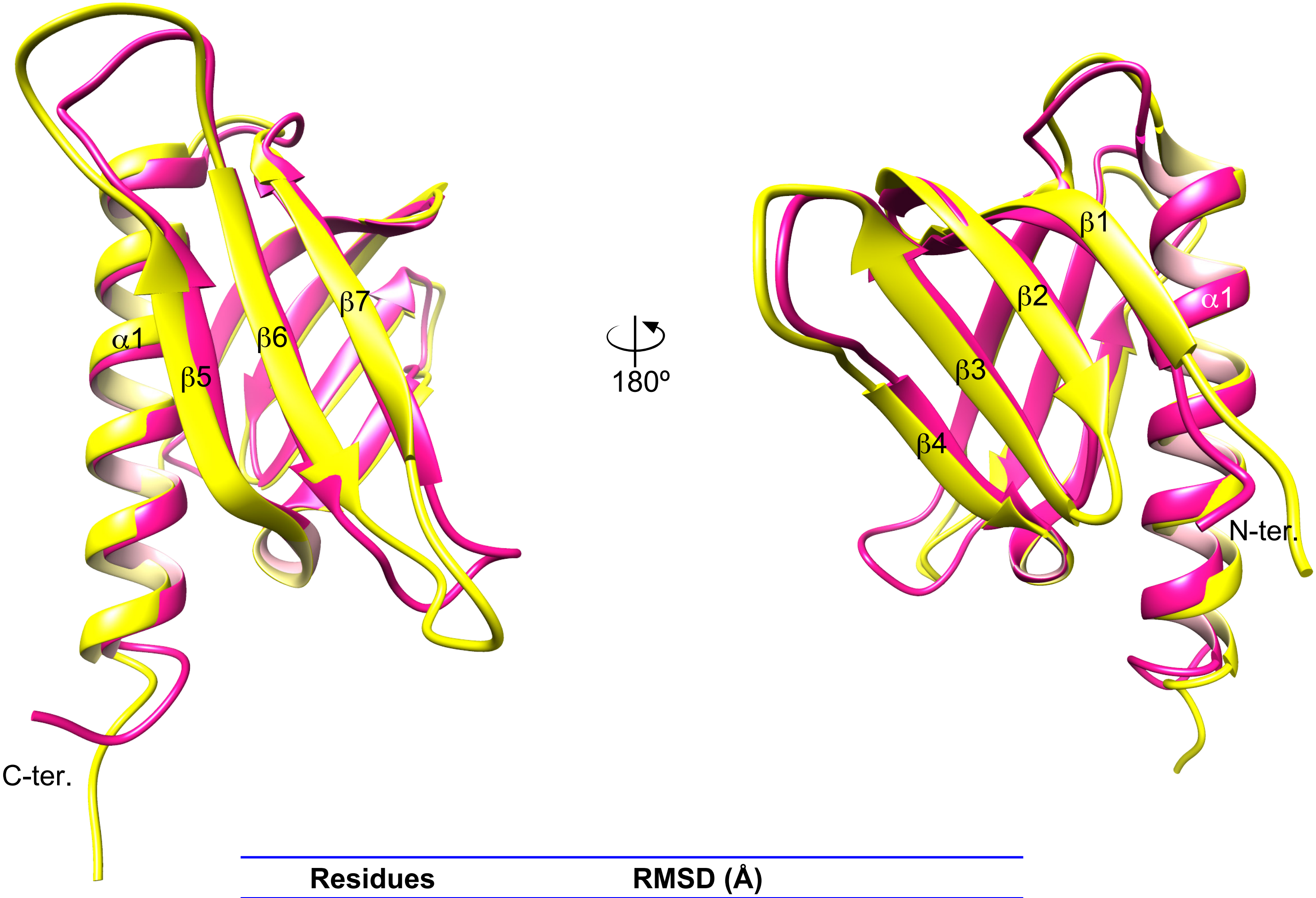

Residues	RMSD (Å)	
4–102	backbone atoms	2.04
	heavy atoms	2.60
4–50, 55–63, 71–102	backbone atoms	1.06
	heavy atoms	2.00

Supplementary Figure 2. Comparison of the NMR structure and AlphaFold structure of spPH domain.
The NMR structure is colored deep pink, the AlphaFold [47] structure yellow. RMSDs are indicated below.

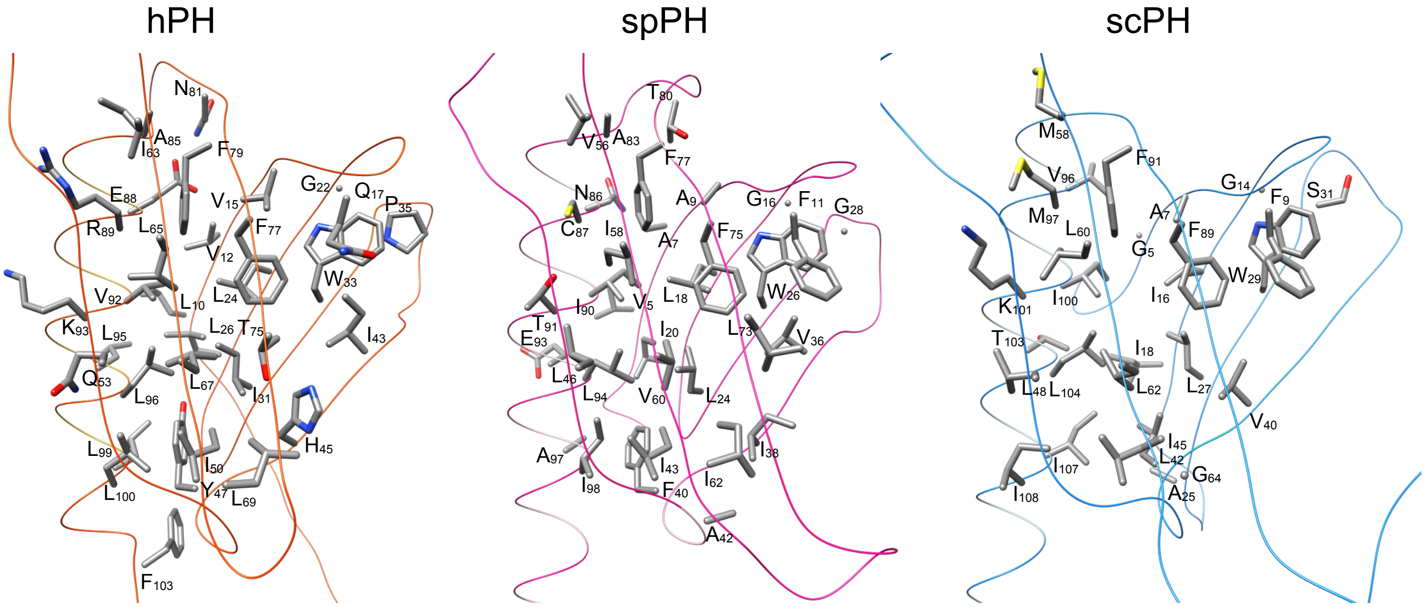

spPH vs hPH

RMSD = 1.05 Å

Identity = 17.5%

spPH vs scPH

RMSD = 1.38 Å

Identity = 31.6%

hPH vs scPH

RMSD = 1.36 Å

Identity = 21.1%

Hydrophobic core regions

Location	hPH	spPH	scPH
β1	L10	V5	(H3)
	V12	A7	G5
	V15	A9	A7
	Q17	F11	F9
β2	G22	G16	G14
	L24	L18	I16
	L26	I20	I18
			A25

Location	hPH	spPH	scPH
β3	I31	L24	L27
	W33	W26	W29
	P35	G28	S31
β4	I43	V36	(H38)
	H45	I38	V40
3_{10}	Y47	F40	L42
	(D49)	A42	(T44)
β5	I50	I43	I45
	Q53	L46	L48

Location	hPH	spPH	scPH
	I63	V56	M58
β6	L65	I58	L60
	L67	V60	L62
	L69	I62	G64
β7	T75	L73	(H87)
	F77	F75	F89
	F79	F77	F91
	N81	T80	

Location	hPH	spPH	scPH
α1	A85	A83	(N93)
	E88	N86	V96
	R89	C87	M97
	V92	I90	I100
	K93	T91	K101
	L95	E93	T103
	L96	L94	L104
	L99	A97	I107
	L100	I98	I108
	F103	(Q101)	(Y111)

Hydrophobic regions

hPH	spPH	scPH
10 – 13	5 – 8	3 – 6
15 – 17	9 – 11	7 – 9
22 – 26	16 – 20	14 – 18
31 – 35	24 – 28	27 – 31
43 – 45	36 – 38	38 – 40
47 – 49	40 – 42	42 – 44
50 – 53	43 – 46	45 – 48
63	56	58
65 – 69	58 – 62	60 – 64
75 – 79	73 – 77	87 – 91
85 – 103	83 – 101	93 – 111

Supplementary Figure 3. Comparison of the hydrophobic core of the hPH, spPH, and scPH domains. Pairwise RMSD and amino acid sequence identity are indicated. Residues that form the hydrophobic core and are present in hydrophobic regions are listed below. Corresponding residues that are not critically involved in formation of the hydrophobic core are indicated in parentheses and not shown on the structure.

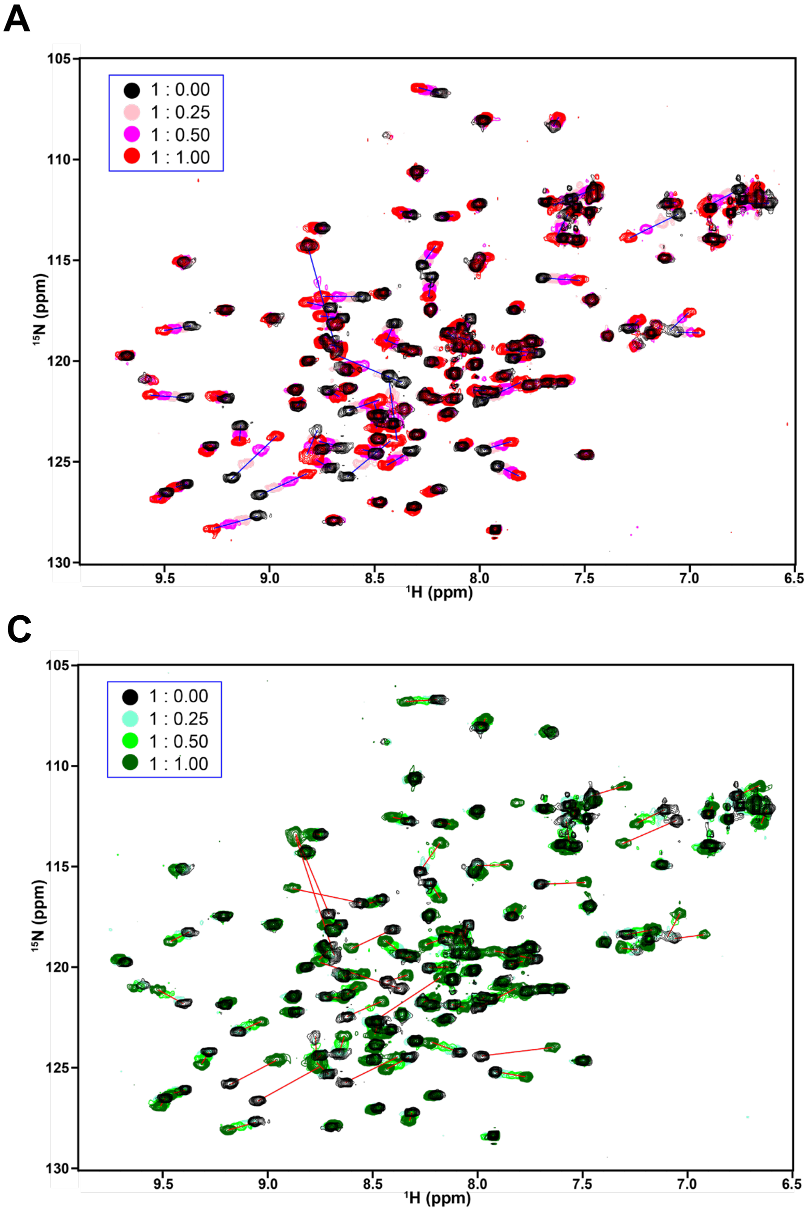

Supplementary Figure 4. Binding analysis of the interactions between the spPH domain and spTfa1$_{332–350}$, spTfa1$_{416–434}$, and spRhp41$_{4–22}$ peptides.
(**A**–**C**) NMR CSP experiment. Overlay of four ^{1}H–^{15}N-HSQC spectra of the spPH domain alone (black) and titrated with target peptide at molar ratios of 1:0.25, 1:0.50, and 1:1.00: spTfa1$_{332–350}$ (**A**); spTfa1$_{416–434}$ (**B**); and spRhp41$_{4–22}$ (**C**).

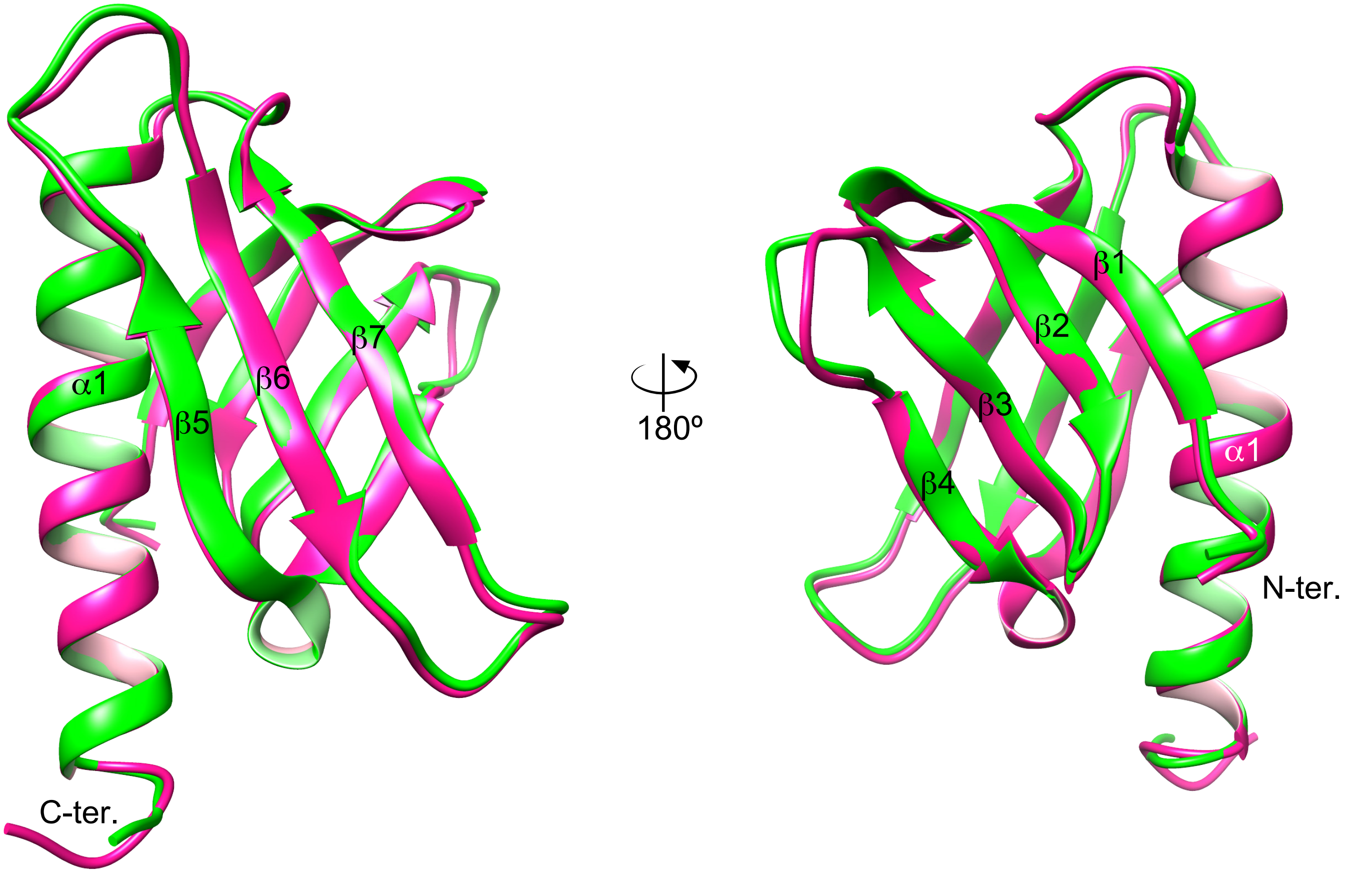

Residues	RMSD (Å)	
4–102	backbone atoms	0.38
	heavy atoms	0.52
4–50, 55–63, 71–102	backbone atoms	0.37
	heavy atoms	0.52

Supplementary Figure 5. Comparison of the NMR structures of the spPH domain calculated with and without using the hydrogen bond restraints.
The structure calculated with the hydrogen bond restraints is colored deep pink, the structure without restraints green. RMSDs are indicated below.
